# Utilization of insecticide-treated nets and associated factors among childbearing women in Northern Nigeria

**DOI:** 10.1186/s12936-023-04620-4

**Published:** 2023-06-16

**Authors:** Bola Lukman Solanke, Daniel Alabi Soladoye, Ibrahim Adamu Birsirka, Anifat Abdurraheem, Omowumi Romoke Salau

**Affiliations:** 1 Department of Demography and Social Statistics, Obafemi Awolowo University, Ile-Ife, Nigeria; 2 Department of Sociology, Bayero University, Kano, Nigeria; 3 Department of Sociology, Federal University, Dutsin-ma, Nigeria; 4 Department of Clinical Nursing Services, UHD Trust, Royal Bournemouth Dorset, Bournemouth, UK

**Keywords:** Insecticide-treated nets, Mosquito bed net, Malaria transmission, Northern Nigeria

## Abstract

**Background:**

Studies have explored the correlates of insecticide-treated nets in Nigeria. The few studies that focused on Northern Nigeria mostly examined individual correlates, but largely ignored the community correlates. Also, the persistence of armed insurgencies in the region calls for more research attention. This study examines the utilization and the associated individual and community factors of insecticide-treated nets in Northern Nigeria.

**Methods:**

The study adopted a cross-sectional design. Data were extracted from the 2021 Nigeria Malaria Indicator Survey (NMIS). A weighted sample size of 6873 women was analysed. The outcome variable was the utilization of insecticide-treated nets. The explanatory variables selected at the individual/household level were maternal age, maternal education, parity, religion, sex of head of household, household wealth, and household size. The variables selected at the community level were the type of place of residence, geo-political zone of residence, the proportion of children under five who slept under a bed net, the proportion of women aged 15–49 who heard malaria media messages, and the community literacy level. Two variables, namely, the number of mosquito bed nets in the household, and the number of rooms used for sleeping were included for statistical control. Three multilevel mixed-effect regression models were fitted.

**Results:**

The majority of childbearing women (71.8%) utilized insecticide-treated nets. Parity and household size were the significant individual/household characteristics associated with the utilization of insecticide-treated nets. The proportion of under-five children in the community who slept under mosquito bed nets, and the geopolitical zone of residence were significant community correlates of the use of insecticide-treated nets. In addition, the number of rooms for sleeping, and the number of mosquito bed nets in the households were significantly associated with the utilization of insecticide-treated nets.

**Conclusion:**

Parity, household size, number of sleeping rooms, number of treated bed nets, geo-political zone of residence, and proportion of under-five children sleeping under bed nets are important associated factors of the utilization of insecticide-treated nets in Northern Nigeria. Existing malaria preventive initiatives should be strengthened to target these characteristics.

## Background

The transmission of malaria is endemic in most low-and-middle-income countries [1–3]. In Nigeria, more than two-thirds of Nigerians are living in communities that are prone to high transmission of malaria with the primary vector being *Anopheles gambiae, Anopheles arabiensis,* and *Anopheles funestus* [4–6]. Global malaria statistics reveal that in 2020, Nigeria accounted for 27 percent of global malaria cases, as well as nearly a quarter of global malaria deaths [7]. In addition, national estimates also showed a significant geographic, urban–rural, and socio-economic disparity in the prevalence of malaria and in the utilization of malaria preventive interventions in the country [8, 9]. Amidst these disparities, malaria transmission continues to have adverse effects on many Nigerians, particularly under-five children, and pregnant women [10–13], which has led to the development and execution of some malaria preventive interventions across the country.

The malaria preventive interventions being implemented seek not only to achieve malaria control but also to ensure the elimination of malaria in the country. These preventive interventions rest largely on the scaling-up of existing preventive initiatives, such as Indoor Residual Spraying (IRS), the universal coverage of Insecticidal-Treated Nets (ITN), and the use of Intermittent Preventive Treatment in pregnancy (IPTp) [14]. Evidence abounds that the use of these preventive interventions is not universal in most parts of the country, particularly among children and childbearing women who are most affected by malaria transmission [15–18]. This has undermined the achievement of malaria control and elimination in the country. The question of whether Nigeria is winning the malaria war posed over six years ago remains unanswered [19]. New evidence indicated that Nigeria remains one of the countries that are not on track to reduce both malaria incidence and deaths by half in 2023 in line with the targets of member nations of the Commonwealth [20].

One key factor that occasioned the lack of universal utilization of malaria preventive interventions in the country is a poor understanding of the associated factors of the preventive interventions. Though, numerous studies have explored the correlates of different malaria preventive interventions in Nigeria and other countries [21–25]. However, studies in Nigeria have insufficiently focused on Northern Nigeria. The few studies that focused on Northern Nigeria mostly examined individual-level correlates but largely ignored the community-level correlates [19, 26–28]. Also, the persistence of armed insurgencies, such as Boko Haram and Islamic State West Africa Province insurgencies, armed banditry, and kidnapping in the region calls for more research attention on Northern Nigeria. This is because the evidence in the literature suggests different links between armed conflicts and malaria transmission [29–31]. These links manifest in at least three possible ways.

One, armed insurgency, such as the Boko Haram insurgency, destroys existing health facilities by reducing the numbers of healthcare personnel, the availability of essential drugs for malaria treatment, hinders access to appropriate care and makes the practice of preventive measures difficult [31–33]. Two, armed insurgency usually causes environmental disruption that not only encourages the breeding of malaria vectors but also hinders larval source management [34, 34]. Three, the movement of government military forces in and out of the battlefronts increases the risk of malaria spread and infection among soldiers who may transmit the same to members of their households [35]. These possibilities do not only adversely affect women’s and children’s health outcomes in the communities affected by armed conflicts but also significantly alter health-seeking behaviour in the affected communities [36–38].

The objective of the study was, therefore, to examine the prevalence and the associated individual and community factors of insecticide-treated nets in Northern Nigeria. The focus is on insecticide-treated nets because among the dominant preventive interventions in Nigeria, it is more widely accessible to Nigerian households, and freely distributed to households during malaria sensitization campaigns [39, 40]. Though some studies have reported challenges in the use of insecticide-treated nets [41], many other studies have established their effectiveness in preventing malaria transmission [42, 43].

The findings in the study will provide additional inputs for developing appropriate strategies to boost the use of insecticide-treated nets in Northern Nigeria. The study was guided by the question: what are the associated factors of the utilization of insecticide-treated nets in Northern Nigeria? The socio-ecological model developed by McLeroy et al*.* [44] anchored the theoretical focus of the study. This model asserts that behavior affects, and is equally affected by multiple factors at different levels. By this logic, the utilization of insecticide-treated nets is affected by the characteristics and behaviour of individuals and communities, which in turn affects the prevalence of malaria in the community. Many existing studies have applied the socio-ecological model to provide an understanding of malaria health-seeking behaviour in different countries [45, 46].

## Methods

### Design and data source

The study adopted a cross-sectional design. The design entails analysing the 2021 Nigeria Malaria Indicator Survey (NMIS) data to describe the malaria-related characteristics of childbearing women in Northern Nigeria and to examine the possible associations between the characteristics and the use of insecticide-treated nets in the region. The survey was executed by the National Malaria Elimination Programme (NMEP) under the auspices of the Federal Ministry of Health (FMoH), and in collaboration with the National Population Commission (NPC) [47]. The general objective of the 2021 NMIS was the provision of valid estimates of malaria-related indicators for the country. Participants in the survey were selected through a two-stage sampling procedure. In the first stage, 568 enumeration areas (used as clusters) were randomly selected. The enumeration areas were based on the frame developed for the forthcoming 2023 population and housing census in Nigeria. The listing of households in the clusters was carried out. In the second stage, households were selected based on a systematic sampling technique. Detailed methodology of the 2021 NMIS has been published elsewhere [47] but could be accessed via https://dhsprogram.com/pubs/pdf/MIS41.pdf. Overall, interviews were completed with 14,476 women aged 15–49 in 13,727 households. The individual and household datasets of the 2021 NMIS were merged in the study because some of the variables of interest were not available in the individual dataset. However, some women were excluded from the study. These are women from the southern region, women who do not have a mosquito bed net for sleeping, and women practicing traditional or other faiths, who were excluded due to their insignificant proportion in the population covered. The study thus analysed a weighted sample size of 6,873 women.

### Outcome variable

The outcome variable in the study is the utilization of insecticide-treated nets. The utilization was measured by asking women whether they slept under a mosquito bed net the night preceding the survey. This yielded a binary response, with ‘yes’ indicating that the respondent utilized the intervention, and ‘no’ indicating otherwise. Existing studies have applied this measure to operationalize the utilization of insecticide-treated nets [9, 15, 48, 49].

### Explanatory variables

The explanatory variables were selected at the individual/household and community levels based on findings in existing studies that the utilization of preventive interventions is influenced by different factors operating at different levels of the social environment, particularly at the individual/household, and community levels [48–51]. The variables selected at the individual/household level were maternal age (15–24, 25–34, and 35 +), maternal education (none, primary, secondary, and higher), parity (nulliparity-no child, primiparity-one child, multiparity-2-4 children, and grand multiparity-5 or more children), religion (Christianity or Islam), sex of head of household (male or female), household wealth (poorest, poorer, middle, richer, and richest), and household size (small or large). These variables have been found to be important correlates of the utilization of malaria preventive interventions in many existing studies [15, 52–56].

The variables selected at the community level were the type of place of residence (urban or rural), geo-political zone of residence (north-central, north-east, and north-west), the proportion of under-five children in the community who slept under mosquito bed nets (low, middle, and high), the proportion of women age 15–49 exposed to malaria media messages in the community (low, middle, and high), and community literacy level (low, middle, and high). These variables have also been identified as important correlates of the utilization of malaria preventive interventions [9, 43, 50, 57]. With the exclusion of type of place of residence and geo-political zone of residence, other community-level variables were generated from individual responses by first aggregating the responses at the cluster level, and then dividing the resulting distribution into three parts of low (0–33%), middle (34–66%), and high (67 + %). Two variables were selected for statistical control. These are the number of mosquito bed nets in the households (0–2, 3–5, and 6 +), and the number of rooms used for sleeping (1–3, 4–7, and 8 +). These variables were previously examined in existing studies [54, 56]. The need for the control variables in the modelling was to further ensure that there is no spurious association among the research variables as their inclusion may affect the significance of some variables.

### Data analysis

Statistical analyses were performed at different levels using Stata version 14 [58]. At the first level, sample characteristics and the utilization of the preventive intervention were presented using frequency distribution and percentages. At the second level, two different analyses were performed. On one hand, the unadjusted binary logit regression coefficients with a 95% confidence interval were used to assess the association between the outcome variable and each of the explanatory variables. This reveals the direction of the relationship (positive or negative) between the variables. Any variable not showing significant association was excluded from further analyses. On the other hand, the Variance Inflation Factor (VIF) was assessed to detect collinear variables. Any variable with a VIF score of ten or higher values was dropped from further regression analyses in line with statistical principles [59]. Variables were then selected for fitting a multilevel mixed-effect regression model, which consists of fixed and random effects [60, 61]. Existing studies that focused on more than the individual-level characteristics also applied multilevel analyses [43, 48–50]. The adjusted Odds Ratio (aOR) with a 95% confidence interval was used to assess the fixed effect of the multilevel model, while the Intra-Cluster Correlation Coefficient (ICC) was used to determine the significance of the community-level variables. The individual/household level characteristics were examined for significance in the fixed-effect component of the model, while the community-level characteristics were examined for significance in the random-effect component of the model. The model adequacy was checked by the Akaike Information Criterion (AIC), which is a widely utilized tool in model selection [62]. Lower AIC values indicate a better fit for the regression model. In addition to the empty model, three other models were fitted. Model 1 included only the individual/household characteristics, while Model 2 controlled for the community-level characteristics. Model 3 was the full model, which included all the research variables. The analyses took into consideration the effect of the NDHS design by applying the DHS weights and using the Svy STATA command to reduce the effects of stratification and clustering.

## Results

### Univariate results

Table 1 presents the respondents’ socio-demographic profiles. As shown in the table, the majority of respondents (71.8%) utilized insecticide-treated nets. Younger women aged 15–24 were dominant in the sample (40.2%), but the proportion of respondents in other age groups was substantial. Half of the sample did not attain any formal education, however, more than a quarter (27.5%) of respondents attained secondary education. Parous women compared to nulliparous women were dominant in the sample, with the highest proportion of parous women having five or more children (33.7%). The majority of the women were Muslims (80.8%). The majority of the household heads were male (93.3%). Respondents from the lower wealth groups outnumbered respondents from the higher wealth groups. Women from large-size households were more than half of the respondents (59.1%), with slightly more than two-fifths of respondents living in small-size households.

**Table 1:** Socio-demographic profile of respondents

Characteristic	Number of Women	Percent	Characteristic	Number of Women	Percent
Malaria Preventive Intervention			Household size		
Utilized	4937	71.8	Small	2810	40.9
Otherwise	1936	28.2	Large	4063	59.1
Maternal age			Number of mosquito bed nets		
15–24	2760	40.2	0–2	3252	47.3
25–34	2373	34.5	3–5	2857	41.6
35 +	1740	25.3	6 +	764	11.1
Maternal education			Number of sleeping rooms		
No education	3433	49.9	1–3	4649	67.6
Primary education	1045	15.2	4–7	2020	29.4
Secondary education	1886	27.5	8 +	204	3.0
Higher education	509	7.4	Region		
Parity			North central	1292	18.8
Nulliparity	1778	25.9	North east	1868	27.2
Primiparity	668	9.7	North west	3713	54.0
Multi parity	2112	30.7	Place of residence		
Grand multiparity	2315	33.7	Urban	1864	27.1
Religion			Rural	5009	72.9
Christianity	1,320	19.2	Proportion of under 5 children who slept under bed net		
Islam	5553	80.8	Low	2193	31.9
Sex of household head			Medium	2130	31.0
Male	6414	93.3	High	2550	37.1
Female	459	6.7	Proportion of women exposed to malaria media messages		
Household wealth index			Low	2308	33.5
Poorest	1738	25.3	Medium	2389	34.8
Poorer	1824	26.5	High	2176	31.7
Middle	1478	21.5	Community literacy level		
Richer	1152	16.8	Low	2450	35.6
Richest	681	9.9	Medium	2281	33.2
			High	2142	31.2
Total	6873	100.0	Total	6873	100.0

Authors’ analysis based on 2021 Nigeria Malaria Indicator Survey

Respondents from the north-west geo-political zone constitute more than half of the women (54.0%), while women from the north-central geo-political zone were the least sampled (18.8%). The majority of respondents reside in rural areas (72.9%). More than one-third of respondents live in communities with a high proportion of children under 5 sleeping under a mosquito net compared to those who reside in communities with either a low or moderate proportion of under 5 children who slept under a mosquito bed net. The proportions of low, medium, or high exposure to media messages about malaria were similar in the communities although the proportion residing in communities with moderate exposure was higher among the respondents (34.8%). The proportion of respondents who reside in communities with low literacy levels was higher among the sampled women (35.7%). Nearly half of the respondents live in households with two or fewer mosquito bed nets (47.3%). Likewise, more than two-fifths of the respondents live in households that possessed three to five mosquito bed nets. The households of the majority of respondents had three or fewer rooms for sleeping (67.6%).

### Bivariate results

Table 2 presents the nature (magnitude and direction) of the relationship between the variables. As evident in the table, maternal age and utilization of insecticide-treated nets were significant (p < 0.01) and positively associated with a consistent increase in the level of utilization as maternal age increased. Maternal education reveals a mixed relationship with the use of insecticide-treated nets. At the lower levels of education, the relationship was positive but at the secondary and higher educational levels, the level of utilization declined consistently showing a negative relationship. Parity and utilization of insecticide-treated nets were positively related showing consistent increases in the level of use as parity of the women increased. Religion and the sex of the head of household and the use of insecticide-treated nets were negatively related, although these were without statistical significance (p > 0.05). Household wealth had a mixed relationship with the utilization of insecticide-treated nets. In the lower and middle wealth groups, a consistent rise in the level of use was observed, but in the richer and richest wealth groups, the level of utilization declined significantly (p < 0.01). Household size and use of insecticide-treated nets were negatively associated, with higher use in small-sized households compared to large-sized households.

**Table 2:** Unadjusted regression coefficient showing the nature of bivariate relationship

Characteristic	% Utilized	β	95% CI	Characteristic	% Utilized	β	95% CI
Maternal age				Region			
15–24^RC^	40.2	–	–	Northcentral ^RC^	18.8	–	–
25–34	34.5	0.29***	0.12–0.46	Northeast	27.2	0.62***	0.37–0.88
35 +	25.3	0.39***	0.20–0.57	Northwest	54.0	0.54***	0.31–0.78
Maternal education				Type of place of residence			
None ^RC^	49.9	–	–	Urban^RC^	27.1	–	–
Primary	15.2	0.05	− 0.18–0.28	Rural	72.9	0.28**	0.07–0.50
Secondary	27.5	− 0.21**	− 0.40, − 0.02	Proportion of Under-5 children who slept under bed net			
Higher	7.4	− 0.48***	− 0.51–0.88	Low^RC^	31.9	–	–
Parity				Medium	31.0	0.69***	0.44–0.94
Nulliparity^RC^	25.9	–	–	High	37.1	1.10***	0.87–1.32
Primiparity	9.7	0.66***	0.42–0.89	Proportion of women exposed to malaria media messages			
Multi parity	30.7	0.69***	0.52–0.87	Low^RC^	33.5	–	–
Grand multiparity	33.7	0.70***	0.51–0.88	Medium	34.8	0.04	−0.21–0.29
Religion				High	31.7	− 0.06	− 0.34–0.22
Christianity^RC^	19.2	–	–	Community literacy level			
Islam	80.8	− 0.03	− 0.24–0.19	Low^RC^	35.6	–	–
Sex of household head				Medium	33.2	0.03	− 0.24–0.29
Male ^RC^	93.3	–	–	High	31.2	− 0.29**	− 0.55, − 0.02
Female	6.7	− 0.10	− 0.38, − 0.18	Number of mosquito bed net			
Household wealth index				0–2^RC^	47.3	–	–
Poorest ^RC^	25.3	–	–	3–5	41.6	0.49***	0.26–0.71
Poorer	26.5	0.11	− 0.13, − 0.35	6 +	11.1	0.87***	0.51–1.22
Middle	21.5	0.16	− 0.10, − 0.41	Number of sleeping rooms			
Richer	16.8	− 0.31**	− 0.58, − 0.03	1–3^RC^	67.6	–	–
Richest	9.9	− 0.54***	− 0.84, − 0.28	4–7	29.4	-0.30***	− 0.49, − 0.11
Household size				8+	3.0	− 0.82***	− 1.39, − 0.25
Small ^RC^	40.9	–	–				
Large	59.1	− 0.53***	− 0.69, − 0.37				

*RC* Reference category

**p < 0.05

***p < 0.01

The association between the proportion of under-five children who slept under a bed net in the community and the use of insecticide-treated nets by childbearing women was positive, with a consistent rise in the level of use as the proportion of children who slept under a bed net in the community increased. In contrast, the proportion of reproductive-age women exposed to media messages about malaria reveals a mixed association with the use of insecticide-treated nets. Likewise, community literacy levels had a mixed association with the utilization of insecticide-treated nets. At the low and moderate literacy levels, a slight rise in the level of utilization was observed, but a dip occurred at the high literacy level. The geopolitical zone of residence had a positive association with the use of insecticide-treated nets, with a higher level of use observed in the northeast zone. A higher level of use of insecticide-treated nets was observed in the rural areas compared to the urban areas showing a significant positive association. A significant positive association was observed between the number of mosquito bed nets and their utilization, while the number of rooms used for sleeping was negatively associated with the use of insecticide-treated nets. All the variables showed a VIF value of less than ten and were selected for the multilevel modeling. However, religion, sex of the head of household, and proportion of women exposed to media messages about malaria were excluded having shown no statistical significance at the bivariate level.

### Multivariable results

Table 3 presents the fixed and random effects on the utilization of insecticide-treated nets. Model diagnosis revealed that Model 3 is the optimal fitted model because it has the minimum value of AIC. The ICC value of the empty model (ICC = 0.335) indicated that in the absence of any of the explanatory variables, a substantial variation in the utilization of insecticide-treated nets was observed across the communities. But in the subsequent model (Model 1), more than one-third of the variation in the use of insecticide-treated nets was attributable to variation in the community-level characteristics (ICC = 0.355). In the model, the majority of the individual/household characteristics had no significant association with the use of insecticide-treated nets. However, while higher parity revealed higher odds of the utilization of insecticide-treated nets, living in the richest household wealth and living in a large-sized household was associated with lower odds of utilizing insecticide-treated nets. With the inclusion of the community-level characteristics in the subsequent model, slight changes were observed in both the source of variation in the outcome variable and the significance of the correlates.

**Table 3:** Measures of association and variation showing effects on utilization of malaria preventive intervention

Characteristic predicting utilization of preventive intervention	Empty Model		Model 1		Model 2		Model 3	
	aOR	95% CI	aOR	95% CI	aOR	95% CI	aOR	95% CI
Maternal age								
15–24^RC^			–	–	–	–	–	–
25–34			1.15	0.88–1.50	1.20	0.92–1.57	1.67	0.90–1.51
35 +			1.21	0.87–1.69	1.31	0.95–1.81	1.24	0.91–1.70
Maternal education								
None^RC^			–	–	–	–	–	–
Primary			1.11	0.82–1.49	1.12	0.83–1.50	1.10	0.83–1.46
Secondary			1.25	0.92–1.70	1.31	0.96–1.78	1.26	0.93–1.69
Higher			1.01	0.63–1.59	1.05	0.66–1.67	0.99	0.64–1.55
Parity								
Nulliparity^RC^			–	–	–	–	–	–
Primiparity			2.60***	1.82–3.71	2.51***	1.76–3.59	2.60***	1.85–3.66
Multiparity			2.77***	2.03–3.73	2.60***	1.92–3.52	2.60***	1.95–3.48
Grand multiparity			3.40***	2.40–4.83	3.17***	2.24–4.50	3.09***	2.21–4.33
Household wealth quintile								
Poorest^RC^			–	–	–	–	–	–
Poorer			1.23	0.88–1.72	1.25	0.90–1.75	1.13	0.83–1.55
Middle			1.32	0.90–1.94	1.54	1.03–2.29	1.39	0.96–2.01
Richer			0.91	0.58–1.42	1.29	0.79–2.10	1.12	0.71–1.77
Richest			0.53**	0.30–0.93	0.86	0.47–1.58	0.73	0.41–1.28
Household size								
Small^RC^			–	–	–	–	–	–
Large			0.34***	0.26–0.44	0.32***	0.25–0.41	0.22***	0.17–0.29
Geopolitical zone of residence								
North-Central^RC^					–	–	–	–
North-East					3.36***	2.14–5.28	2.38***	1.59–3.56
North-West					2.21***	1.43–3.40	1.68***	1.14–2.47
Type of place of residence								
Urban^RC^					–	–	–	–
Rural					1.09	0.74–1.60	1.01	0.71–1.43
Proportion of under-five children in the community who slept under bed net								
Low^RC^					–	–	–	–
Middle					4.11***	2.77–6.12	3.66***	2.57–5.20
High					7.17***	4.63–11.09	5.79***	3.93–8.52
Community literacy level								
Low^RC^					–	–	–	–
Middle					1.23	0.81–1.85	1.23	0.85–1.78
High					1.09	0.64–1.86	1.08	0.67–1.74
Number of mosquito bed nets in the household								
0–2^RC^							-	-
3–5							4.25***	3.28–5.52
6 +							11.47***	7.13–18.45
Number of sleeping rooms in the household								
1–3^RC^							–	–
4–7							0.46***	0.35–0.61
8 +							0.21***	0.10–0.44
Log likelihood	− 3366.2		− 3244.6		− 3179.4		− 3085.8	
AIC	6738.5		6525.3		6412.7		6233.9	
LR test	χ^2^ = 686.0, p < 0.01		χ^2^ = 643.6, p < 0.01		χ^2^ = 448.2, p < 0.01		χ^2^ = 311.3, p < 0.01	
ICC	0.335 (33.5%)		0.355 (35.5%)		0.216 (21.6%)		0.164 (16.4%)	

*RC* Reference category

**p < 0.05

***p < 0.01

In Model 2, higher parity remained significantly associated with higher odds of the use of insecticide-treated nets, while a large household size remained significantly associated with lower odds of the use of insecticide-treated nets. Other individual/household characteristics revealed no statistical significance. Two community-level characteristics, namely, the proportion of under five children who slept under a mosquito bed net in the community and geopolitical zone of residence were significantly associated with the utilization of insecticide-treated nets. However, the proportion of variation in the use of insecticide-treated nets attributable to the community factors dropped compared to the proportion in Model 1 (ICC = 0.216).

In the full model (Model 3), on one hand, two individual/household characteristics, namely, parity and household size, were significantly associated with the use of insecticide-treated nets. On the other hand, two community characteristics, namely, the proportion of under-five children who slept under a mosquito bed net in the community and the geopolitical zone of residence were significant correlates of the use of insecticide-treated nets. As parity was increasing, the odds of utilizing insecticide-treated nets also increased consistently. Childbearing women living in large-sized households were associated with lower odds of utilizing insecticide-treated nets (aOR = 0.22, 95% CI 0.17–29). As the proportion of under-five children who slept under mosquito bed nets in the community increased, the odds of utilizing insecticide-treated nets by childbearing women consistently increased. The odds of utilizing insecticide-treated nets were higher in the northeast zone (aOR = 2.38, 95% CI 1.59–3.56) and northwest zone (aOR = 1.68, 95% CI 1.14–2.47) compared to the northcentral zone. Also, an increasing number of mosquito bed nets in households was associated with higher odds of their utilization. In contrast, increasing the number of rooms for sleeping in households was associated with lower odds of utilizing insecticide-treated nets.

## Discussion

The study examined the prevalence and associated factors of the utilization of insecticide-treated nets in Northern Nigeria. The study found a utilization level lower than the observed average for the region in a recent Nigerian study [49]. However, the disparity in the two results is likely due to the inclusion of more categories of childbearing women in the current study compared to the inclusion of only pregnant women in the earlier study. The prevalence found in the study is much higher than the prevalence reported in another Nigerian study [51]. Though the two studies are based on data obtained from the NMIS. The data analyzed in the current study is however more recent and included larger numbers of reproductive-age women. This may be the source of variations in the two results. Also, the prevalence revealed in the study is much higher than the reported levels in two earlier studies conducted in two different states of Northern Nigeria [27, 28]. Nevertheless, the current finding confirms that the utilization of insecticide-treated nets is not universal in Northern Nigeria. Improving the current situation requires more proactive steps from all tiers of government in Northern Nigeria. The persistence of armed conflicts in the region makes such proactive steps imperative in the region based on established evidence of the adverse effects of armed conflicts on malaria transmission and healthcare use [31–34, 34–38]. It is noteworthy that the utilization of insecticide-treated nets was observed to be highest in the northwest zone which has been less devasted by armed insurgencies compared to the northeast zones. This tends to suggest either that governments efforts to revive the health delivery system in the north-west zone are yielding quick positive results or that individuals in the zone have improved in personal health-seeking behavior by preventing mosquito transmission in the wake of the disruption of public health delivery system due to insurgencies. This, however, implies that malaria control and elimination initiatives need to be scaled up in the northeast, which has witnessed more incidences of insurgencies and disruption of the public healthcare delivery system.

The study further revealed that some individual/household and community characteristics are important correlates of the utilization of insecticide-treated nets. One, gender matters in the utilization of insecticide-treated nets. It is reasoned that since more females compared to males use malaria preventive interventions [27], women are likely to extend use to their successive newborns and under-five children. Besides, women stay at home with children more than men and are more likely to ensure that their newborns and under-five children sleep under the bed net. This provides support for the increasing calls for mainstreaming gender into malaria control and elimination activities [63]. Two, large household sizes may be a barrier to utilizing insecticide-treated nets. The reason for this finding may not be unconnected with insufficient numbers of treated bed nets available in the households. As found in the study, nearly half of the households had two or fewer treated bed nets, which is not likely to be sufficient for the use of all household members. Bearing in mind that the study location is a region with widespread poverty, especially among women, the majority of the people in the region are likely to rely on the free distribution of treated bed nets for their use. This calls for the scaling-up of the free distribution of treated bed nets across the nooks and crannies of Northern Nigeria to enhance its utilization in the region.

Three, community contexts are source of variations in the level of use of insecticide-treated nets, though not in a substantial proportion. These community factors include the geo-political zone of residence and the proportion of under-five children in the community who slept under treated bed nets. The geopolitical zone of residence, particularly being residents in the states in the northeast and northwest zones was found to the drive utilization of insecticide-treated nets in line with findings in two recent studies [50, 51]. This may not be unconnected with the persistence of armed insurgencies in the two zones, which may have created the conditions for malaria transmission to thrive in the zones. In boosting the utilization of malaria preventive interventions in the two zones, it is necessary to get communities to key into malaria control and elimination programs of governments and non-governmental organizations through more community-based engagements. Such engagements could be through mobilizing community leaders and associations to amplify national and regional malaria control targets with the view of encouraging more use of the treated bed nets in the communities.

## Conclusion

The paper assessed the prevalence and correlates of the utilization of insecticide-treated nets in Northern Nigeria based on data extracted from a nationally representative survey. The study revealed that the utilization of insecticide-treated nets was not universal among childbearing women in the region and that some individual-level and community-level characteristics were associated with the utilization of insecticide-treated nets. Improving the level of utilization in the region requires targeting more individual characteristics of childbearing women, as well as embarking on more community engagements to amplify governments and non-government strategies to boost utilization of malaria preventive measures.

## Data Availability

Secondary data analyzed could be accessed online at https://dhsprogram.com/data/.

## Abbreviations

FMoH: Federal Ministry of Health
IPTp: Intermittent preventive treatment of malaria in pregnancy
IRS: Indoor residual spraying
LLINs: Long-lasting insecticidal nets
NMIS: Nigeria malaria indicator survey
NPC: National population commission
WHO: World Health Organization
NMSP: National malaria strategic plan
